# Clinical Society of New York Policlinic Medical School and Hospital

**Published:** 1912-01

**Authors:** 


For Texas Medical Journal.

### Clinical Society of New York Policlinic Medical School and Hospital.

Meeting December 5, 1911.

Case of tubercular iritis, presented by Dr. E. S. Thompson:

The case was interesting as one of primary tubercular iritis. The patient was an Italian, 21 years of age, two years in the country. He has three brothers living and well, and one sister, who died at the age of 7. No family history of tuberculosis. He reacted positively to the Von Piquet test, but otherwise shows no evidence of tuberculosis.

*Eye Symptoms.*—Besides the usual adhesions of the lense, he shows a well marked grayish nodule, projecting from the periphery of the iris, which is rather characteristic. The three growths on the iris which it is most important to distinguish are sarcoma, so-called gumma, and tubercular.

Sarcoma is usually a dark distinct mark, which is characteristic; gumma is usually on the margin, and is associated with a good deal of inflammation, but clears up very rapidly under mercury. Tubercle most frequently occurs at the limbus. In this case one can see a cheesy mass, extending from the limbus to the adjacent structures. With the Von Piquet reaction and the clinical appearance, it seems a fair clinical conclusion that it is tubercle of the iris.

These cases are generally secondary to some process of the lung, and the primary cases that have been reported are open to question. The prognosis in this case appears favorable and the patient will probably recover if properly treated.

In response to a query as to what treatment would be effective, Dr. Thomson said he had been impressed with the value of tuberculin, and would start that treatment immediately. He would take the precaution, however, of having a Wasserman test made. Surgical treatment was not indicated.

Dr. Lynch said he supposed that the same principles would hold good in this case as in other types of T. B., viz., good hygienic surroundings and good food.

Report of a case of cancer of the stomach, presented by Dr. Kellog:

Dr. Kellog had operated upon this case several days before and, although the patient was doing well in the ward, regretted that he could not show it.

The man was about 50 years of age. About three years ago after business anxiety he complained of pain in the stomach during the earliest stages of digestion, and attacks of vomiting. Later the time of the pain changed. He was awakened with pain in the night. After a time these symptoms disappeared.

Two months ago there was a recurrence of symptoms; he had some tenderness and pain, located in the gall bladder region. The pain occurred before he had entered the hospital, coming on four to four and a half hours after food. The vomitus was dark in color, and foul smelling. He had no night pain during this attack. After reaching the hospital the night pain came on and suggested duodenal location. Examination of stomach contents showed excessive HCl, faint trace of lactic acid, and a few Boas bacilli.

The X-ray showed an interruption of the peristaltic wave at one point. Physical examination of the stomach showed no enlargement and no tumor could be felt. From an examination of the stomach contents and the X-ray findings, an exploratory incision was suggested. Operation disclosed a mass on the posterior portion of the stomach.

The interesting features of the case were the total absence of physical signs and the establishment of the diagnosis by the X-ray findings. A gastro-enterostomy was performed successfully.

Dr. Lynch inquired what preparation of bismuth was used.

Dr. Kellog said the pictures were taken by Dr. Busby, who uses a preparation of bismuth in buttermilk. He did not know whether subnitrate or subcarbonate was used.

Dr. Grant said he expected from the history of the case to find duodenal ulcer, the period which came so long after eating being suggestive. Examination of gastric contents he has not found of much value in cases of duodenal ulcer. The delayed period of pain is, however, of importance. It is claimed that increase of HCl is a characteristic of the condition. In several of his cases there was no increase, while in others there was decided diminution in HCl.

Dr. Wightman said that simple ulcers of the stomach required care in the diagnosis, and subsequent treatment, as a malignant condition, frequently became engrafted upon simple ulcers, and were prone to run a very acute course.

Dr. Yeomans emphasized the value of X-ray pictures in carcinoma, as they were extremely helpful in locating the site of the tumor. It facilitated finding the lesion on the operating table when time was valuable.

Dr. Edgerton said that the X-ray might be misleading, as in a case he had recently observed with Drs. Hays, Nisbet and Foote. The X-ray plates showed interference with the peristaltic wave at the lower end of the greater curvature of the stomach, but operation proved a Meckel's diverticulum in the ileum near the cecum.

Dr. Lynch said that he did not think any particular method should be depended upon entirely, but that all should be taken together, not omitting the X-ray or gastric content examination. It had been observed that subnitrate in quantity may have poisonous effect, for that reason Kirks and others have adopted the chloride of bismuth, which is harmless and suspends very easily in milk.

Dr. Lynch gives two ounces of chloride of bismuth suspended in kumyss at night, the bowels having been emptied by an enema, and the next morning two ounces more, and thus combined, the X-ray picture gives both stomach and colon.

This may be done every three or four days for four or five pictures preceding operation until the desired picture is secured.

